# Reconstruction of Large-scale Defects with a Novel Hybrid Scaffold Made from Poly(L-lactic acid)/Nanohydroxyapatite/Alendronate-loaded Chitosan Microsphere: *in vitro* and *in vivo* Studies

**DOI:** 10.1038/s41598-017-00506-z

**Published:** 2017-03-23

**Authors:** Hongwei Wu, Pengfei Lei, Gengyan Liu, Yu Shrike Zhang, Jingzhou Yang, Longbo Zhang, Jie Xie, Wanting Niu, Hua Liu, Jianming Ruan, Yihe Hu, Chaoyue Zhang

**Affiliations:** 1 Department of Orthopedics, Hunan Cancer Hospital and The Affiliated Cancer Hospital of Xiangya School of Medicine, Central South University, Changsha, 410013 Hunan China; 2 Department of Orthopedics, Xiangya Hospital, Central South University, Changsha, 410011 China; 3 Department of Orthopedics, Brigham and Women’s Hospital, Harvard Medical School, Boston, Massachusetts 02115 United States; 4 Department of Orthopedics, The Third Xiangya Hospital, Central South University, Changsha, 410013 China; 5 Biomaterials Innovation Research Centre, Department of Medicine, Brigham and Women’s Hospital, Harvard Medical School, Cambridge, Massachusetts 02115 USA; 6 Department of Neurosurgery, Xiangya Hospital, Central South University, Changsha, 410011 China; 7 Department of Neurosurgery, School of Medicine, Yale University, New Haven, Connecticut USA; 8 Department of Orthopedics, VA Boston Healthcare System, Boston, MA USA; 9 Dr. Li Dak Sum & Yip Yio Chin Center for Stem Cell and Regenerative Medicine, School of Medicine, Zhejiang University, Hangzhou, 310058 China; 10 Powder Metallurgy Research Institute, Central South University, Changsha, 410083 Hunan China

## Abstract

A chitosan-based microsphere delivery system has been fabricated for controlled release of alendronate (AL). The present study aimed to incorporate the chitosan/hydroxyapatite microspheres-loaded with AL (CH/nHA-AL) into poly(L-lactic acid)/nanohydroxyapatite (PLLA/nHA) matrix to prepare a novel microspheres-scaffold hybrid system (CM-ALs) for drug delivery and bone tissue engineering application. The characteristics of CM-ALs scaffolds containing 10% and 20% CH/nHA-AL were evaluated *in vitro*, including surface morphology and porosity, mechanical properties, drug release, degradation, and osteogenic differentiation. The *in vivo* bone repair for large segmental radius defects (1.5 cm) in a rabbit model was evaluated by radiography and histology. *In vitro* study showed more sustained drug release of CM-AL-containing scaffolds than these of CM/nHA-AL and PLLA/nHA/AL scaffolds, and the mechanical and degradation properties of CM-ALs (10%) scaffolds were comparable to that of PLLA/nHA control. The osteogenic differentiation of adipose-derived stem cells (ASCs) was significantly enhanced as indicated by increased alkaline phosphates (ALP) activity and calcium deposition. *In vivo* study further showed better performance of CM-ALs (10%) scaffolds with complete repair of large-sized bone defects within 8 weeks. A microspheres-scaffold-based release system containing AL-encapsulated chitosan microspheres was successfully fabricated in this study. Our results suggested the promising application of CM-ALs (10%) scaffolds for drug delivery and bone tissue engineering.

## Introduction

Critical-sized bone defects are a common clinical condition generally caused by trauma, bone disease, or tumor resection^1^. The consequent bone loss cannot be repaired physiologically and bone regeneration in large quantities is generally required^2^. Bone grafting, such as autografts and allografts, has been commonly used for bone induction and augmentation in large bone defects^3^. However, these bone grafts have some limitations that make synthetic bone graft substitutes an attracting alternative^4, 5^.

An ideal bone graft substitute for tissue engineering application should serve as both a drug delivery system and an osteoconductive scaffold that coordinately enhance tissue regeneration^6^. The biodegradable synthetic polymers, such as Poly L-lactic acid (PLLA), poly(lactic-co-glycolic acid) (PLGA), and Poly (ε-Caprolactone) (PCL), have been used to construct porous scaffolds for tissue engineering^7–9^. Poly(L-lactic acid) (PLLA)-based scaffold is a synthetic bone graft substitute that has been largely investigated for bone tissue engineering applications^10, 11^. Although both the PLLA and PLGA have been approved by the FDA and have been successflly used as orthopaedic implants for tissue engineering^7, 9^. PLLA scaffolds have been reported to show better biodegradable and mechanical properties than 50:50 poly(lactic-co-glycolic acid) (PLGA), and Poly (ε-Caprolactone) (PCL) scaffolds, along with the improved bone ingrowth and mineral deposition^12^. The degradation time was suggested to vary with the ratio of lactic acid and glycolic acid polymer^13, 14^. Besides, previous studies of biomineral coatings on Solid Freeform Fabricated PLLA and PCL Scaffolds indicted that PLLA scaffolds showed more bone ingrowth than PCL scaffolds. Biomineral coatings enhanced bone ingrowth in both the PLLA and PCL scaffolds, while there were still more mineralized tissue formation in the coated PLLA scaffolds than that of the coated PCL scaffolds after implantation^8, 15^. Therefore, PLLA were selected as the scaffold material in our study. The nano-hydroxyapatite (nHA), a bioactive and biocompatible compound accepted for use in bone tissue engineering, has been further dispersed to provide additional mechanical strength and integrity. HA incorporation could not only improve the cell activity and viability, mechanical properties of the scaffolds, and buffer the acidic degradation products from the polyester^16, 17^, but also impart osteoconductivity to the PLLA/HAP porous scaffolds and improve the protein adsorption capacity of the scaffolds^18, 19^. The PLLA/nHA matrices have been suggested to be an ideal scaffold in tissue engineering^20^, especially for large bone defect healing^21^. However, osteoinductive materials are often required for bone regeneration of the scaffolds.

Alendronate (AL) is a potent inhibitor of bone resorption and has been primarily used as the first-line therapy for osteoporosis^22^. It may exert antiresorptive and osteoanabolic effects through inhibiting osteoclastic activity and inducing osteoblast anabolic activity^23^. Recent studies have indicated that AL stimulated the expression of bone morphogenetic protein 2 (BMP2), a key factor involved in osteogenic differentiation and bone regeneration^24, 25^, and enhanced osteogenesis of human adipose-derived stem cells (ASCs) as well as bone repair of critical-sized calvarial defects^26^. Induction of osteogenic differentiation by AL^26, 27^ makes it a promising candidate for bone tissue engineering. However, low bioavailability of AL in aqueous conditions makes up the major limitation for its utilization. This limitation may be overcome by hybridizing it with other materials^28, 29^, which in turn resulted in an increased loading efficiency and a sustained release of AL for a long time^30, 31^. Chitosan is a natural biomaterial that has been widely used in biomedical engineering as pharmaceutical excipient for controlled drug delivery^32^. Our previous study has fabricated the emulsion cross-linked chitosan/nano-hydroxyapatite microspheres for AL delivery (CH/nHA-AL), and the promising properties of the prepared microsphere implicated it as a promising candidate for local treatment of bone defects^33^. Therefore, in the present study, our previously constructed CH/nHA-AL was incorporated into the PLLA/nHA matrix to prepare a novel microspheres-scaffold-based release system (CM-ALs) for drug delivery and bone tissue engineering application. The *in vitro* characteristics and osteogenic difference of the prepared scaffold system were studied. Furthermore, the *in vivo* bone regenerative capability was investigated in a rabbit model of critically-sized segmental bone defects.

## Results

### Characterization of PLLA/nHA/CM-AL scaffolds

#### Morphology and porosity of the scaffolds

**Figure 1:**
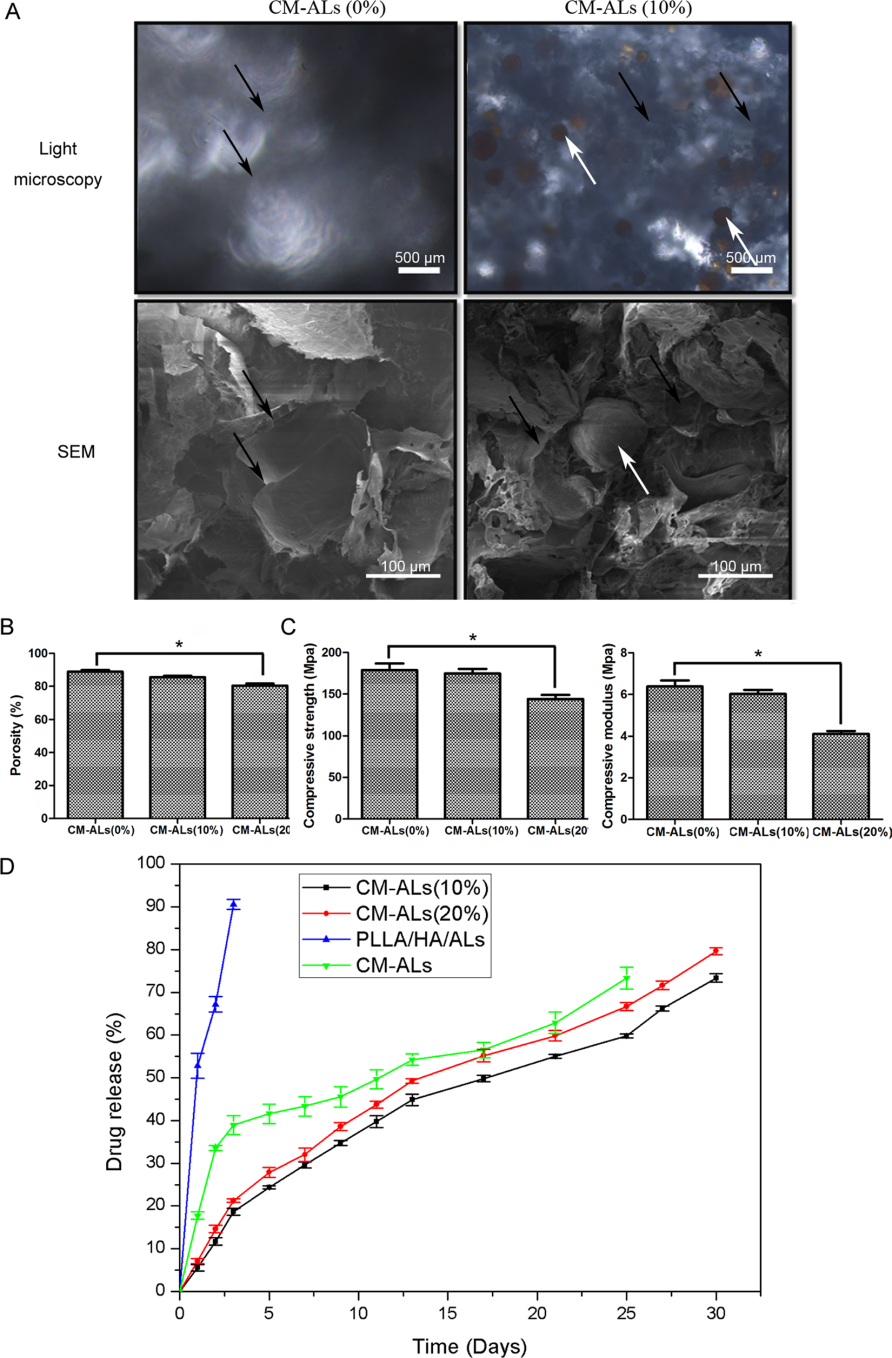
Characteristics of CM-ALs scaffolds containing different concentrations of CH/nHA-AL. (**A**) Morphology of CM-ALs (0%), CM-ALs (10%) and CM-ALs (20%) scaffolds under light microscopy (LM) and scanning electron microscopy (SEM); (**B**) Porosity of CM-ALs scaffolds with different CH/nHA-AL contents; (**C**) Compressive strength and modulus of CM-ALs scaffolds with different CM-ALs contents; (**D**) *In vitro* drug release assay of CM-ALs scaffolds for a period of up to 25 days using CH/nHA-AL and PLLA/nHA/AL scaffolds as control. Black arrow indicates PLLA/nHA matrix, while white arrow indicates CH/nHA-AL microsphers. *p < 0.05 vs CM-ALs (0%) scaffolds.

The morphology features of the scaffolds were visualized by light microscopy and SEM as shown in Fig. 1. The cross-section analysis of scaffolds under light microscopy showed that photic zones and CH/nHA-AL (brown) were uniformly distributed in the scaffolds (Fig. 1A). The scaffolds exhibited a homogeneously interconnected porous structure under SEM, with the pore diameters of 150–250 μm. The morphology of scaffolds was quite different between the two scaffolds (PLLA/nHA and CM-AL) with CH/nHA-AL partially embedded in the CM-AL scaffolds (Fig. 1A).

The porosity of the scaffolds was detected by liquid replacement approach. The porosity of 88.90 ± 2.15% was obtained in the PLLA/nHA scaffolds. Addition of 10% CH/nHA-AL resulted in a porosity of 85.51 ± 1.96%. This further decreased to 80.38 ± 2.784% with CH/nHA-AL contents increasing to 20%, which was significantly lower than that of the other two groups (p < 0.05, Fig. 1B).

### Mechanical properties of the scaffolds

The mechanical properties of CM-ALs scaffolds were evaluated. The compressive strength and modulus decreased with the introduction of 10% CM-ALs. However, the data were not statistically significant from that of the PLLA/nHA control (89.35 ± 9.07 vs 87.35 ± 6.23 MPa, and 3.19 ± 0.32 vs 3.01 ± 0.21 MPa, respectively). However, increasing the CH/nHA-AL content to 20% resulted in a significantly poorer mechanical properties when compared with that of the other two groups (72.02 ± 5.36 MPa and 2.06 ± 0.15 MPa, p < 0.05, Fig. 1C).

### *In vitro* drug release of the scaffolds

The drug release profile was characterized using CM-ALs and PLLA/nHA/AL scaffolds as control. The PLLA/nHA/AL scaffolds showed an initial burst release of AL within the first 3 days (90.57 ± 1.16%). CM-ALs scaffolds exhibited a much slower release, with the sustained release of AL for a period of up to 25 days (Fig. 1D). Although the drug release of the scaffolds was controlled with the addition of CH/nHA-AL, there was no significant difference between the scaffolds containing the different contents of CM-ALs (10% and 20%).

### *In vitro* degradation of the scaffolds

**Figure 2:**
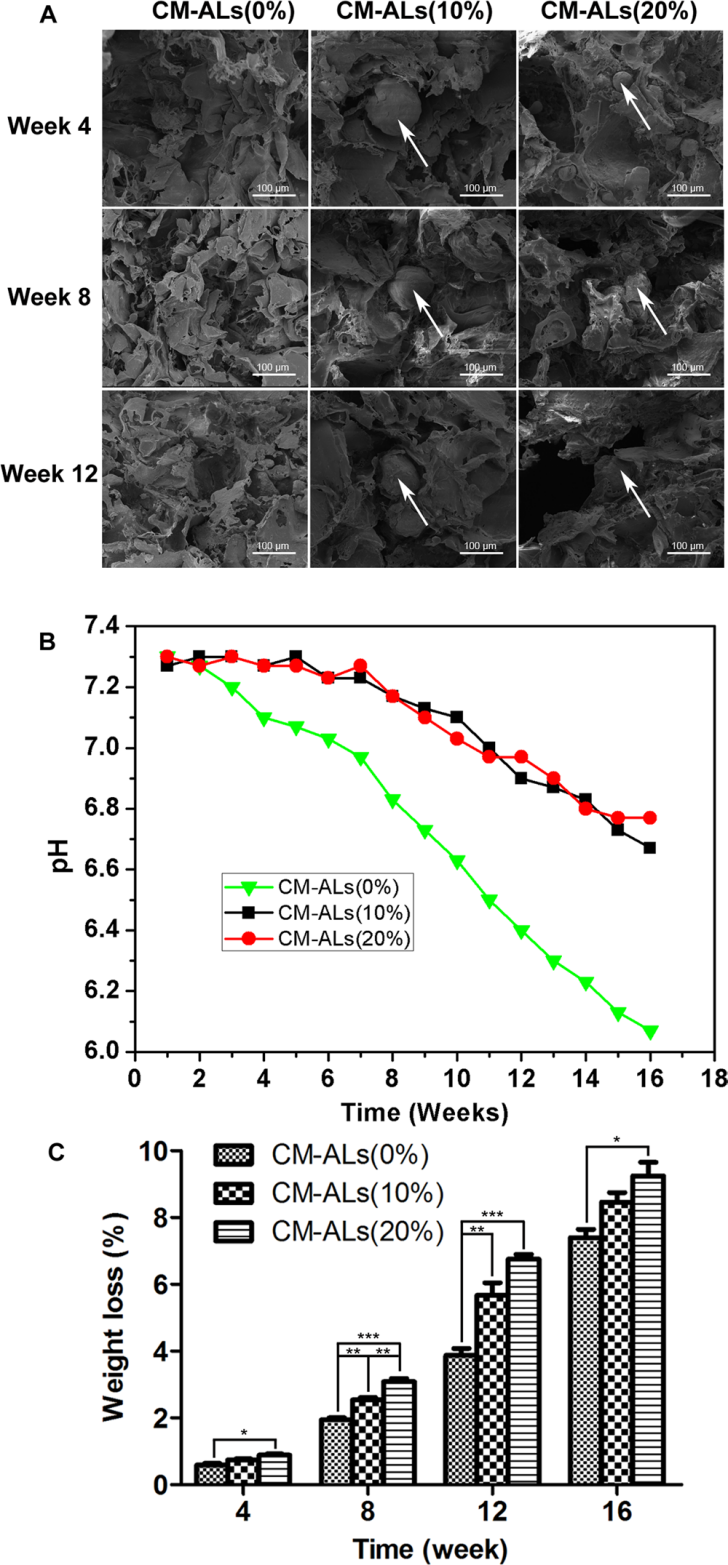
*In vitro* degradation of the CM-ALs scaffolds with different CH/nHA-AL contents during the 12 weeks of observation. (**A**) Morphological changes of the scaffolds observed under SEM; (**B**) pH value of degradation medium from the prepared scaffolds with the increasing incubation time; (**C**) Mass loss of the scaffolds during the degradation period. White arrow indicates CH/nHA-AL microsphers. *p < 0.05, **p < 0.01, ***p < 0.001 vs CM-ALs (0%) or CM-ALs (10%) scaffolds.

The dissolution behaviors of prepared scaffolds were assessed by morphological observation, pH variation, and weight loss. The morphological changes of the scaffolds were analyzed by SEM. The scaffolds containing 20% of CH/nHA-AL showed obvious degradation, while degradation of the CM-ALs (10%) scaffolds proceeded slower during the 12 weeks of observation (Fig. 2A). The pH variations of degradation medium were evaluated during the process. Degradation of PLLA/nHA scaffolds significantly decreased the pH value of medium, while a lower variation was observed in CH/nHA-AL-containing scaffolds, with the constant higher values obtained during the 16 weeks of incubation. There was no significant difference in pH variation between the scaffolds containing 10% and 20% of CH/nHA-AL (Fig. 2B). Weight loss of the scaffolds increased with the increased CH/nHA-AL contents, and the procedures proceeded slowly in CH-ALs scaffold during the whole degradation period (Fig. 2C).

In view of the drug release, degradation and mechanical properties, CM-ALs scaffolds containing 10% of CH/nHA-AL (CM-ALs (10%)) were used for the following study.

### *In vitro* biocompatibility of scaffolds

#### Cell Morphology and Adhesion on scaffolds

**Figure 3:**
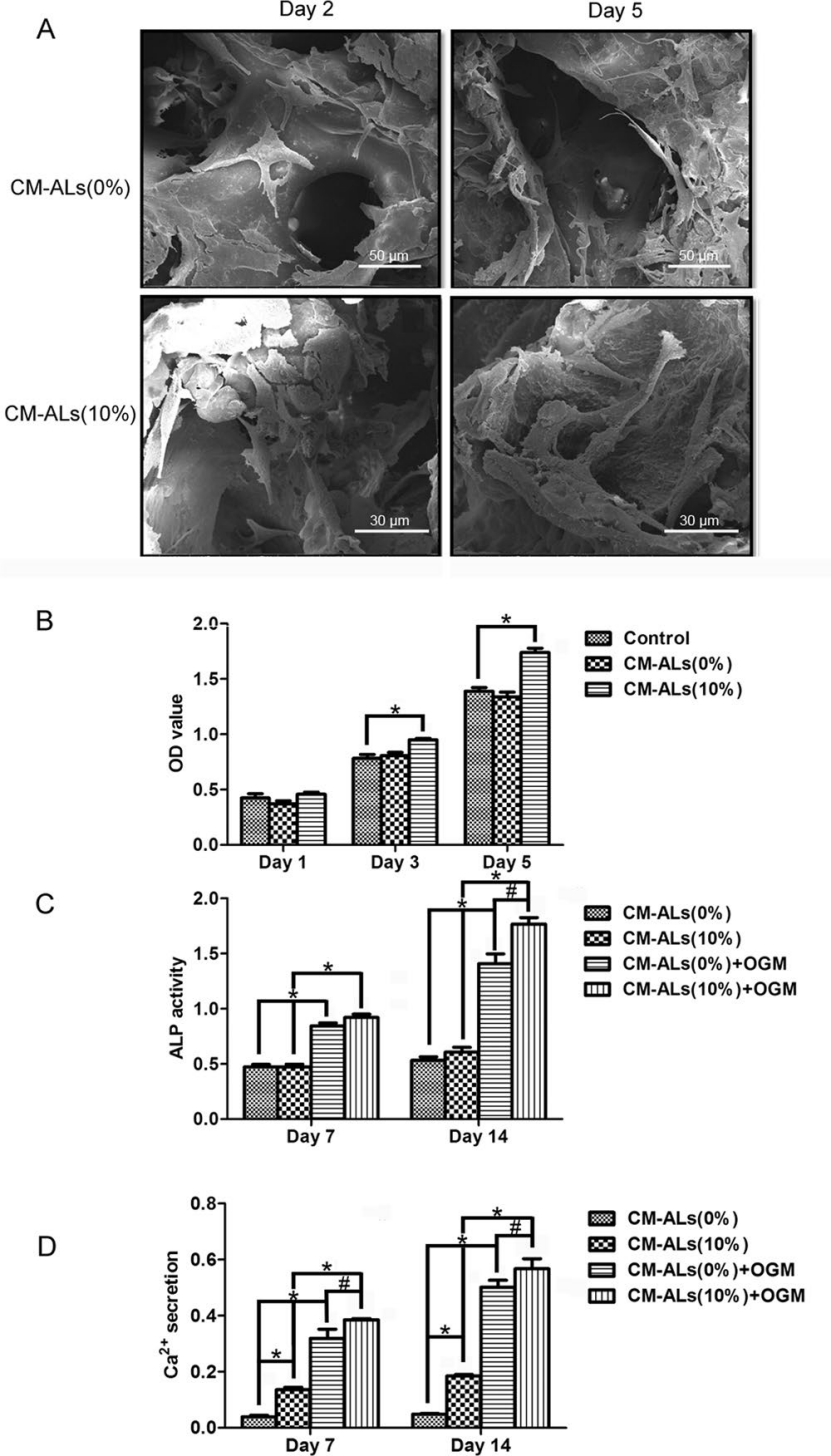
*In vitro* cytocompatibility of the CM-ALs scaffolds. (**A**) Cell morphology and adhesion of rabbit ASCs on scaffolds under SEM; (**B**) Cytotoxicity analysis of ASCs survived in scaffold leaching liquor using MTT assay; (**C**) Alkaline phosphate (ALP) activity of ASCs in scaffold leaching liquor; (**D**) Mineralized matrix synthesis was analyzed by Alizarin Red staining for calcium deposition. White arrow indicates CH/nHA-AL microsphers. *p < 0.05 vs CM-ALs (0%) scaffolds; ^#^p < 0.05 vs CM-ALs (0%) scaffolds in present of OGM.

Cell attachment and proliferation on the scaffolds were evaluated as shown in Fig. 3. Light micrographs showed long spindle-like cells spread on the scaffolds, with clear cytoplasm and smooth surfaces. The numbers of rabbit ASCs on the scaffolds increased dramatically over a period of 3 to 5 days. The SEM analysis showed elongated stellate cells attached on the scaffolds with multiple filopodia. After culture of ASCs for 5 days, the cells were proliferating in the interconnected pore channel. The filopodia elongation was observed with extracellular matrix actively secreted (Fig. 3A).

#### *In vitro* cytotoxicity of scaffolds

MTT assay was performed to determine cytotoxicity of the extracted liquid from the prepared scaffolds. As shown in Fig. 3B, extracted liquids from the both scaffolds (PLLA/nHA and CM-ALs (10%)) showed no apparent cytotoxicity, and viability of cells increased with incubation time of 5 days. However, rabbit ASCs incubated in the extracted liquids from CM-ALs (10%) scaffolds showed higher viability than those in the PLLA/nHA scaffolds group on incubation days 3 and 5.

#### *In vitro* osteogenesis of ASCs on the scaffolds

To assess the osteogenesis of rabbit ASCs induced by controlled release of AL from the scaffolds, ALP and alizarin red staining assays were done at days 7 and 14. As shown in Fig. 3C, extracted liquids from the both scaffolds in DMEM + 10% FBS culture medium did stimulate ALP activity of ASCs on days 7 and 14. In the case of OGM supplement, ALP activities were significantly enhanced, and the effect was further enhanced with the addition of CH/nHA-AL into the PLLA/nHA scaffolds (p < 0.05). Mineralized matrix synthesis was analyzed by Alizarin Red staining for calcium deposition. Calcium deposits of ASCs were significantly induced with the presence of CH/nHA-AL in the scaffolds as well as OGM supplement. The highest value was identified when ASCs were incubated in extracted liquids from the CM-ALs scaffolds that supplied with OGM, and the data were increased with time (Fig. 3D).

### *In vivo* bone regeneration of the scaffolds

The CM-ALs (10%) scaffolds were implanted into a rabbit model of large segmental radius defects to further evaluate the *in vivo* bone regeneration.

#### X-ray analysis of bone regeneration

**Figure 4:**
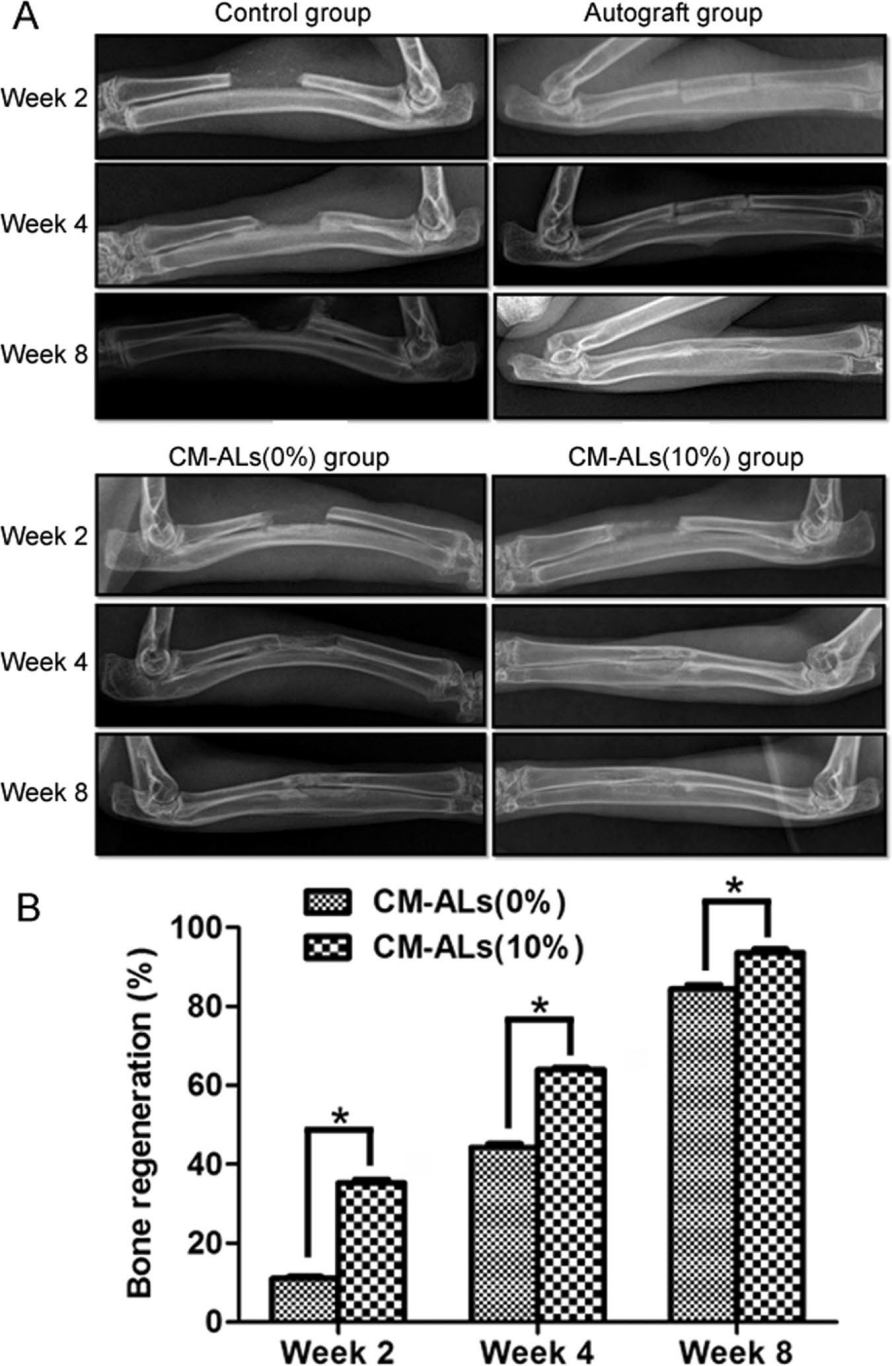
X-ray analysis of bone regeneration at different times after implanting operation. (**A**) Representative radiographs of bone regeneration at different times. In the control group, there was some new bone formation but the bone defect was not repaired; In autograft group, autologous orthotopic transplantation allowed a complete healing of radius defects during the 8 weeks follow-up; In CM-ALs (0%)-implanted group, there was obvious new bone formation at week 8. The bone fracture lines were scarcely observed, while bone marrow cavities were not found to become interconnected; In CM-ALs (10%)-implanted group, bone defects were healed with new bone formation during 4 to 8 weeks. (**B**) Semiquantitative analysis showed significant new bone formation in CM-ALs (10%)-implanted group when compared CM-ALs (0%)-implanted group and data increased with time. *p < 0.05 vs the CM-ALs (0%) control group.

X-ray analysis showed the defects healing during the 8 weeks follow-up (Fig. 4A). In the control group (group I), there was some new bone formation but the bone defect was not repaired due to the large bone defect. In autograft group (group II), autologous orthotopic transplantation allowed a complete healing of radius defects during the process. In CM-ALs (0%)-implanted group (group III), there was a small amount of callus formation at week 2 and increased over time. At week 4, bone regeneration was observed at defected area and large callus formation was seen aligned along the shaft axis. There was obvious new bone formation at week 8. The bone fracture lines were scarcely observed, while bone marrow cavities were not found to become interconnected. In CM-ALs (10%)-implanted group (group VI), the obvious callus formation was noted at week 2. Bone defects were healed with new bone formation during 4 to 8 weeks. The bone outlines were formed on the outer side of bone calluses, and the regenerated bone was well remodeled. Semiquantitative analysis showed significant new bone formation in CM-ALs (10%)-implanted group when compared with CM-ALs (0%)-implanted group and data increased with time (p < 0.05, Fig. 4B).

#### Histological analysis of bone regeneration

**Figure 5:**
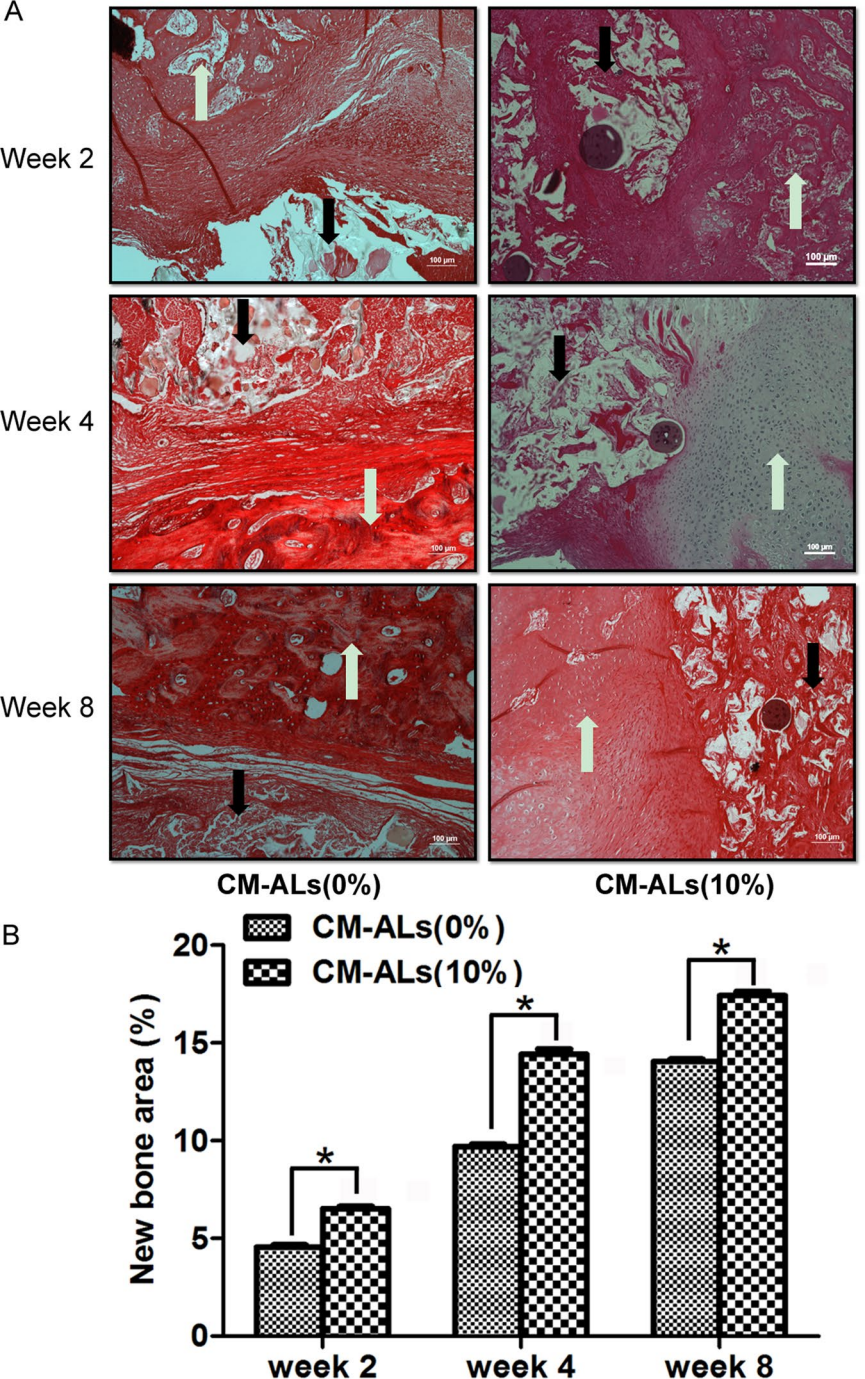
Histological analysis of bone regeneration and scaffold degradation at different times after implanting operation. (**A**) HE staining showing a small amount of newly formed trabeculae at 2 weeks in both CM-ALs (0%) and CM-ALs (10%) groups. At week 4 and 8, coarse bone trabeculae were presented and clustered with the vascular structure clearly seen. The fibrous tissue filled defect was still observable in the CM-ALs (0%) group, while it was less visible in the CM-ALs (10%) group; (**B**) The quantitative analysis showed the increased new bone area with the incorporated CM-ALs and increased days. White arrows indicate areas of new bone formation, while black arrows indicate the scaffolds (Magnification: ×40). *p < 0.05 vs the CM-ALs (0%) control group.

**Figure 6:**
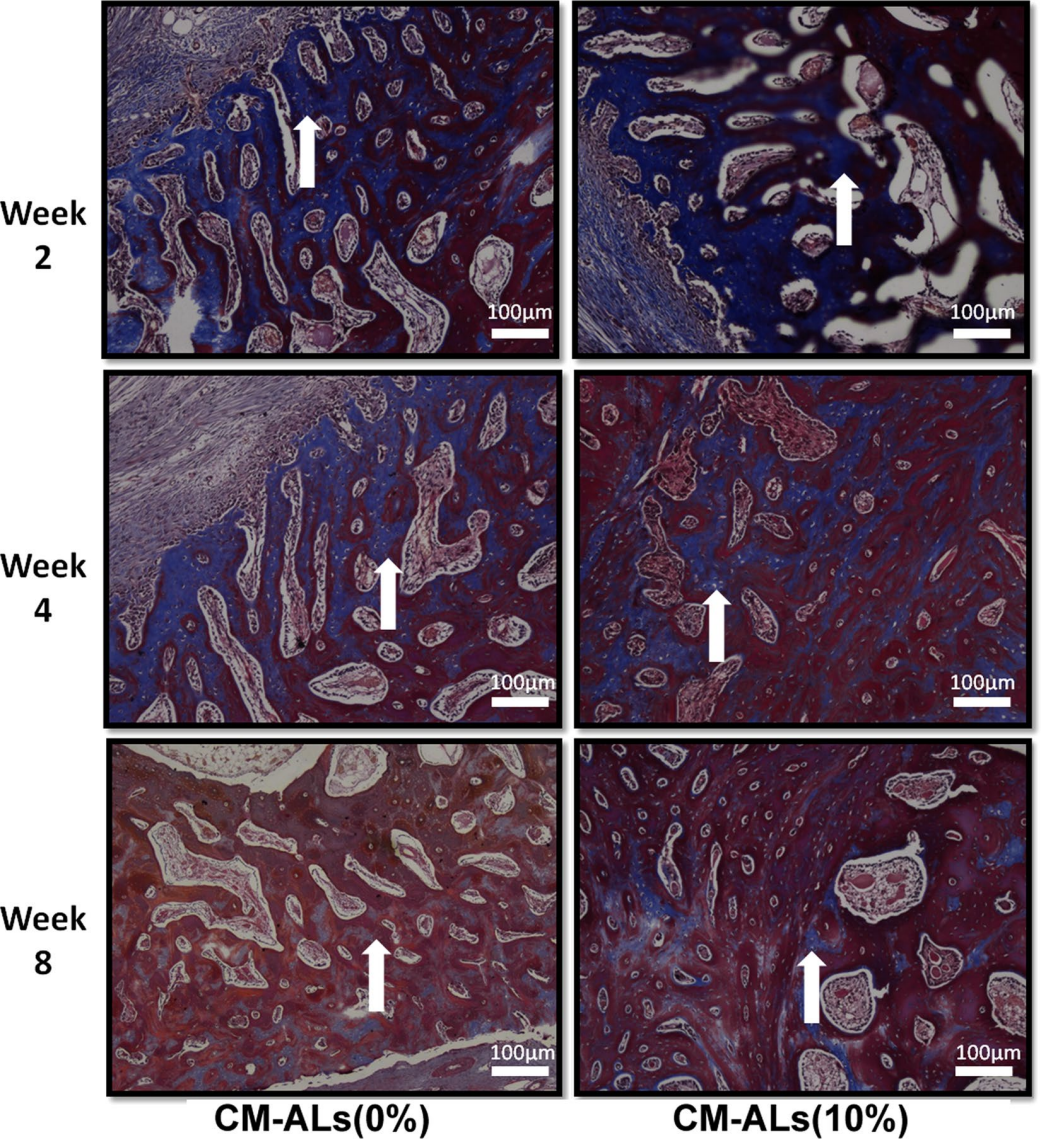
Masson staining showed gradual maturation of the newly formed bone in both groups. Newly formed bone were stained by heavy blue (as white arrow shown) on week 2 and the blue area shrank gradually as bone become more mature on week 8, indicating osteogenesis initiated as early as week 2 in both groups but osteogenesis process lasted longer in CM-ALs (10%) group than in CM-ALs (0%) group (Magnification: ×40).

HE and Masson trichrome stainings were further performed for the histological analysis. As shown in Fig. 5A, a small amount of newly formed trabeculae were seen at 2 weeks in both groups. The defect was filled with fibrous connective tissue with the new blood vessels dispersed. At weeks 4 and 8, coarse bone trabeculae were presented and clustered with the vascular structure clearly seen. In the CM-ALs (0%) group, the fibrous tissue filled the defect was still observable, while it was less visible in the CM-ALs (10%) group. The quantitative analysis showed the increased new bone area with the incorporated CM-ALs and increased days (Fig. 5B). Masson staining showed gradual maturation of the newly formed bone in both groups. Newly formed bone were stained by heavy blue on week 2 and the blue area shrank gradually as bone become more mature on week 8, indicating osteogenesis (collagen fiber synthesis) initiated as early as week 2 in both groups but osteogenesis process lasted longer in CM-ALs (10%) group than in CM-ALs (0%) group (Fig. 6).

## Discussion

Synthetic bone graft substitutes have played an increasingly important role in large bone defect healing. A good initial mechanical stability and optimal interconnected porous architecture provide the major challenge in the development of adequate bone substitutes. PLLA/nHA-based mixture is one of the most common bioresorbable composite bone substitutes available up to now, and it has already been approved for clinical use due to its good biodegradability and biocompatibility^21^. However, osteoinductive materials are often required for effective bone regeneration. Increasing amount of studies have demonstrated the osteogenic effect of AL as a potent osteo-inductive factor and AL loading has been suggested as a promising therapeutic approach^26, 34^. However, AL is highly hydrophilic and can easily dissolve in aqueous conditions, which makes better-controlled drug release systems of critical importance. Chitosan is a favorable natural material that attracts increasing attention for drug delivery and bone tissue engineering due to its favorable biocompatibility and biodegradability^35^. Our previous study has successfully constructed a class of nHA/chitosan-based microspheres for controlled release of AL, with improved loading efficiency and sustained drug release, as well as the enhanced osteogenic activity of rabbit ASCs^33^. Therefore, in this study, these CH/nHA-AL microspheres were further dispersed into PLLA/nHA matrices to construct the composite CM-ALs scaffolds for large bone defect repair. The characteristics of the prepared scaffolds were analyzed. The CM-ALs scaffolds were rectangular in shape. Porosity and mechanical properties are two major parameters for scaffolds of bone tissue engineering. Introduction of 10% of CH/nHA-AL into PLLA/nHA scaffolds did not significantly change the porosity and mechanical properties of the scaffolds. However, further increase of the CH/nHA-AL contents to 20% caused a significant decrease in porosity and mechanical properties of the prepared scaffolds. This finding partially differed from the previous studies, which indicated that the porosity of the PLLA/nHA/CMs composites was only apparently decreased when the CMs contents was increased to 50%, while the compressive strength and modulus in their study increased with the enhanced contents of CMs^36, 37^. These conflict results can be partially explained by particle size distribution of chitosan microspheres, which may contribute to the interconnected PLLA scaffold geometry and thus poor mechanical properties. Added to this is the possible variation in components of the scaffolds, and all of these difficulties may contribute to the controversial results.

Release of AL from porous CM-ALs scaffolds was analyzed using CH/nHA-AL alone and porous PLLA/nHA-AL scaffolds as control. AL was rapidly released from the PLLA/nHA-AL scaffolds with a 90% cumulative release within 3 days. Compared with the fast drug release from the PLLA/nHA-AL scaffolds, the release behavior of AL from the CH/nHA-AL microspheres and CM-ALs scaffolds sustained for up to 25 days. The release profiles of CH/nHA-AL microspheres showed a burst release of AL within the early period. An initial burst release of AL has also been reported in other AL-loaded scaffolds^29, 38–40^. In our study, further incorporation of CH/nHA-AL into PLLA/nHA scaffolds obviously alleviated this burst AL release, and the prepared scaffolds showed more sustained release of AL for up to 30 days. There was no obvious difference in release profile of PLLA/nHA scaffolds with different concentrations of CH/nHA-AL (10% and 20%). These results were dramatically different from the study reported by Park and colleagues, which indicated a concentration-dependent release profile of their AL-loaded biphasic calcium phosphate scaffolds, with the high concentration ones shown more sustained AL release^39^. The cause for these differences may be partially explained by the different bone graft substitutes used, different techniques used for preparation of the scaffolds, and the different AL concentration levels involved. However, despite of these, our study still indicated that CM-ALs scaffolds offered sustained release kinetics for AL delivery.

*In vitro* degradation assay showed the enhanced degradation of CM-ALs scaffolds with the increased CH/nHA-AL contents, and there was no significant degradation difference between CM-ALs (10%) scaffolds and PLLA/nHA control. pH variation was lower during *in vitro* degradation of porous CM-ALs scaffolds than that of PLLA/nHA scaffolds, indicating the degradation of both PLLA matrix and CMs. Meanwhile, the weight loss ratio was significantly increased in CM-ALs scaffolds while mechanical properties were significantly lower. These results were consistent with the previous study, which indicated that weight loss of the PLLA-based composites increased with the enhanced CMs dosage, and it was attributed to a preferential dissolution of the chitosan component in the composites^41^. The rapid degradation of the introduced CMs has been suggested to contribute to the early phase weight loss of the scaffolds^37^. With all these together, our results showed evidence suggesting that PLLA/nHA scaffolds with 10% of CH/nHA-AL showed preferable characteristics, while increased the CH/nHA-AL contents to 20% caused the unfavorable effect. PLLA/nHA scaffolds containing 10% CH/nHA-AL were therefore used in the further studies.

*In vitro* bioactivity of the CH/nHA-AL-containing PLLA/nHA scaffolds was further analyzed. Biocompatibility of the prepared scaffold composite was investigated by seeding rabbit ASCs into the PLLA/nHA and CM-ALs (10%) scaffolds. Adhesion and distribution of ASCs were characterized and the results showed a good Biocompatibility. *In vitro* cytotoxicity assay showed a better proliferation of ASCs on the CM-ALs (10%) scaffolds as compared to PLLA/nHA scaffolds. Cell viability in CM-ALs (10%) scaffolds was significantly higher with increased culture days. This may be attributed to the chitosan component that has been reported to promote cell survival^42, 43^ and AL released. Previous studies of controlled release of AL using the poly (lactic-co-glycolic acid) (PLGA), chitosan, or biphasic calcium phosphate (BCP)-based systems have been conducted for bone repairing applications. Cells grown on these AL-loaded systems showed the enhanced *in vitro* osteogenesis, as demonstrated by higher levels of ALP secretion and/or calcium deposition^26, 29, 30, 38–40^. Therefore, osteogenic differentiation of ASCs was assessed in our study by monitoring ALP activity and calcium deposition at days 7 and 14. ALP activity and calcium deposition were significantly enhanced by maintained ASCs in the released liquid of CM-ALs scaffolds supplied in OGM, when compared with cells incubated in extracted liquids of PLLA/nHA scaffolds supplied with OGM. This should be attributed to the osteogenic differentiation induced by AL, which has been extensively demonstrated to enhance osteogenes^29, 34, 44–46^. All these results suggested that the CM-ALs (10%) scaffold is more suitable for cell survival and proliferation and shows better osteogenic differentiation of ASCs than the PLLA/nHA scaffold.

*In vivo* studies of AL on bone regeneration have been investigated. Local injection of AL on human ASCs seeded PLGA scaffolds by Wang and colleagues were reported to enhance human ASCs osteogenesis and bone regeneration in a rat model of critical-sized (7-mm) calvarial defect, and the maximal effect was observed at week 12^26^. Loading AL on polycarprolactone (PCL) nanofibrous scaffolds by Yun and colleagues has shown a positive effect on bone regeneration in a circular transosseous defect (diameter: 8 mm) at week 8^47^. The recent study by Park *et al*. have investigated the bone regeneration effect of AL-loaded BCP scaffolds, and the enhanced bone formation was observed in a rat segmental diaphyseal tibial defect (7 mm) model. However, their results showed no solid bony bridge formation until week 8^39^. Therefore, the *in vivo* bone regenerative capability of our prepared scaffold composites was further investigated by implanting PLLA/nHA and CM-ALs (10%) scaffolds into a critical-sized bone defect (1.5 cm segment) model of rabbit. The bone regeneration was evaluated by radiographic and histological assessment. CM-ALs (10%) scaffolds group showed excellent bone healing with complete repair of the defects within the 8-week observation period, comparable to that of autograft group. Incorporation of AL-encapsulated CH/nHA microspheres into PLLA/nHA scaffolds resulted in more excellent performance in bone regeneration when compared with the PLLA/nHA scaffolds. Taken together, the *in vivo* study demonstrated a good bone regeneration of our CM-ALs (10%) scaffolds.

However, this study also has some limitations. Primarily, molybdenum blue colorimetry was used for determination of AL concentration based on the phosphate anion in water. Although the method has been demonstrated to be reproducible, accurate and suitable for AL determination^33, 48^. However, distilled water was used in place of PBS that simulates the constant pH of body fluid, since a large amount of phosphate from PBS may interfere with the AL determination. Therefore, other methods that can better simulate body fluid conditions may be considered for AL determination in our future work.

In conclusion, AL-encapsulated CH/nHA microspheres were successfully incorporated into PLLA/nHA based scaffolds, and a microspheres-scaffold-based release system has been developed for AL delivery and bone tissue engineering. CM-ALs (10%) scaffolds showed more sustained drug release and comparable mechanical and degradation properties. The osteogenic differentiation of ASCs was also enhanced as demonstrated by significantly increased ALP activity and calcium deposition. *In vivo* study showed excellent bone healing of CM-ALs (10%) scaffolds, comparable to that of autograft. Therefore, the results of this study suggested the promising application of CM-ALs (10%) scaffolds for drug delivery and bone tissue engineering.

## Material and Methods

### Preparation of PLLA

PLLA were produced by ring-opening polymerization of lactide. Briefly, L-lactide monomer (90%, Jiangxi Wuzangye Biochemical Industry Co., Ltd., Shanghai, China) was dehydrated at 140–150 °C for 4–5 h, followed by decompression dehydration at 2.5–8.0 kPa 170 °C for 6–8 h. The products were depolymerized to lactide under the existence of stannous octoate (0.27 kPa, 37 °C). The lactide was purified by repeated recrystallization using ethyl acetate as the solvent. The ring-opening polymerization was performed to prepare PLLA under the existence of lactide and stannous octoate as initiator and catalyst (8000:1). The PLLA copolymer was characterized by gel permeation chromatography (GPC) and Fourier transform infrared spectroscopy as previously described^49^.

### Preparation of porous CM-ALs scaffolds

**Figure 7:**
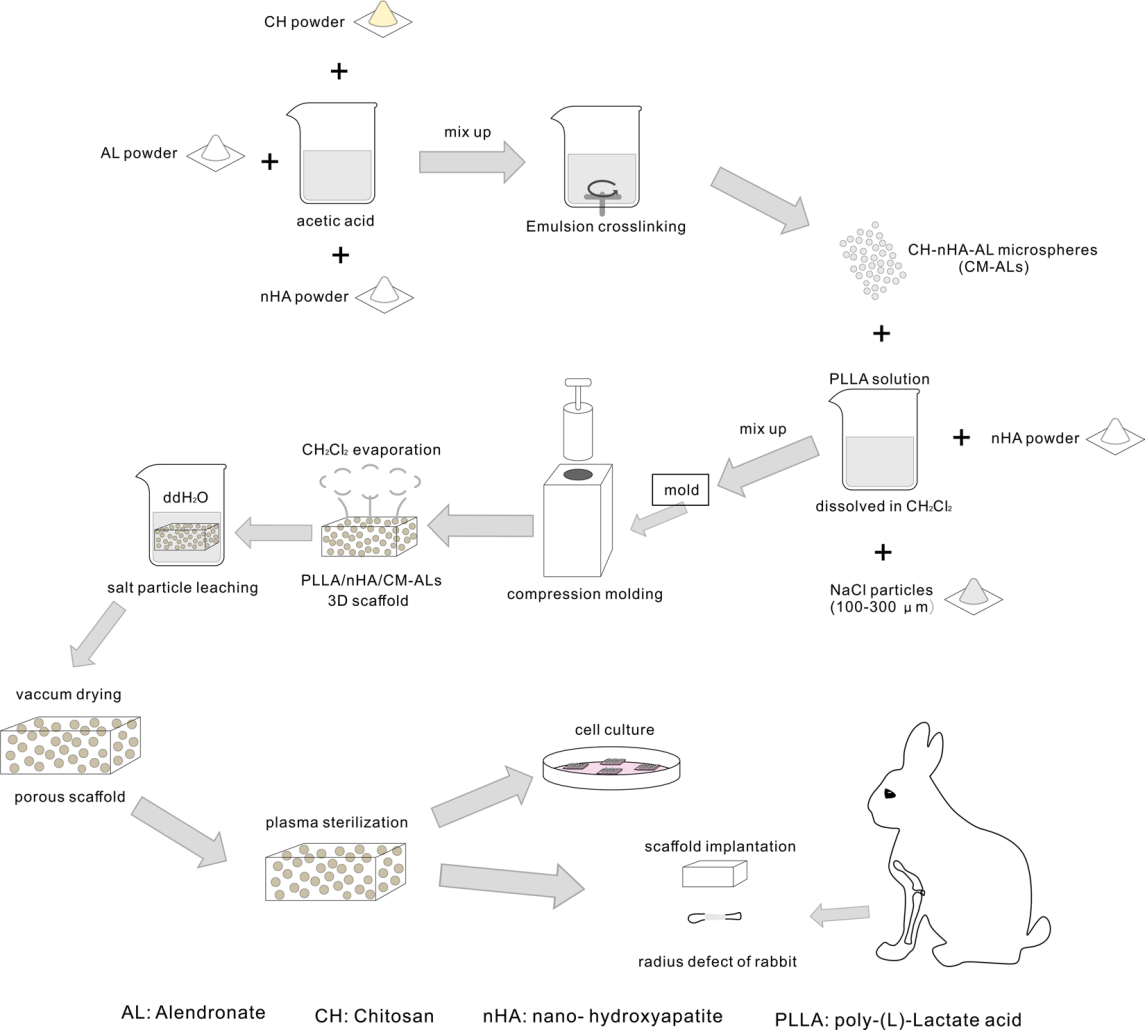
Schematic illustration of preparation of PLLA/nHA/CM-AL scaffolds for bone regeneration in a rabbit model of large segmental bone defects.

The porous CM-ALs scaffolds were prepared using room temperature molding/particle leaching method^50^. PLLA granules were dissolved in dichloromethane (0.05 g/mL) and the bottle was sealed to prevent solvent evaporation. CH/nHA-AL were fabricated as described in our previous report^33^. PLLA, nHA, and CH/nHA-AL were mixed together using an electronic magnetic stirrer for 20 mins. The mixture was stirred further with the bottle open to obtain paste like consistency. The products with a final concentration of 10% and 20% CH/nHA-AL, termed as CM-ALs (10%) and CM-ALs (20%), were fabricated for modulation, respectively. NaCl particles of 150–200 μm were used as pore-forming agent. The mixture was poured into a mold (7 × 7 × 16 mm) and compressed for 3 min at a pressure of 50 N. After molding for 24 h, the moulds were placed in the fume hood for dichloromethane evaporation. The scaffolds were then immersed in deionized water for 36 h, and the water was replaced every 12 h. The scaffolds were then dried in a vacuum drying oven at 40 °C and −0.1 MPa for 24 h. Illustration of the forming process of PLLA/nHA/CM-AL scaffolds and morphology of the prepared scaffolds were shown in Fig. 7. The porous PLLA/nHA scaffolds with only PLLA and nHA were used as control (CM-ALs (0%)).

### Characterization of PLLA/nHA/CM-AL scaffolds

#### Morphology and porosity properties

The morphology of PLLA/nHA/CM-AL scaffolds was visualized by light microscopy (Olympus U-RFL-T, Tokyo, Japan) and scanning electron microscopy (SEM, Nova NanoSEM230, USA). Porosity of scaffolds was measured using Archimedes’ principle as described previously^51^. Briefly, the scaffolds were placed in a graduated cylinder filled with a certain amount of ethanol (V1). The total volume following scaffold immersion (V2) and volume of ethanol left in the cylinder (V3) after scaffolds removal were recorded. The porosity was then determined using the following equation:

$${\rm{Porosity}}\,( \% )=\frac{{\rm{V}}1-{\rm{V}}3}{{\rm{V}}2-{\rm{V}}1}\times 100 \% $$

#### Mechanical properties

The scaffolds of 20 ± 0.7 mm in length and 7 ± 0.5 mm in width were prepared and dried in vacuum for 24 h. The mechanical properties were measured using Instron 1121 universal mechanical testing machine at room temperature, with the loading speed of 1.0 mm/min and mechanical deformation of 2 mm. The compressive modulus was calculated using the DTM-II dynamic elastic modulus tester (Xiangyi Instruments Co., Ltd., Xiangyi, China). Three specimens were tested for each sample.

#### *In vitro* drug release

*In vitro* drug release was measured according to our previous method with some modification^33^. Briefly, each scaffold was placed in a dialysis bag immersed in distilled water (40 mL) and shaken at a speed of 45 rpm at 37 °C for 25 days. Supernatant (2 mL) was collected each day and quantified by molybdenum blue colorimetric assay.

#### In vitro degradation

Rectangular scaffold samples were immersed in phosphate buffer solution (PBS) and monitored by pH variation and weight loss ratio. The pH value was measured once a week for 16 weeks. Weight loss of the scaffolds was detected every 4 weeks after vacuum drying during the degradation period. Three specimens were prepared from each scaffold for each time points.

### *In vitro* biocompatibility assay

#### Cell morphology and adhesion assay

The scaffolds were co-cultured with rabbit adipose-derived stem cells (ASCs, Cyagen Biosciences Inc., Guangzhou, China) for cell morphology and adhesion assay. Scaffolds were cut into small pieces (7 mm × 7 mm × 1 mm) and sterilized using low temperature plasma sterilization. ASCs (1 × 10^4^ cells/mL, 100 μL) were seeded on the scaffolds and incubated in a 37 °C incubator for 2 h to allow for cell attachment. The 2 mL of Dulbecco’s modified Eagle’s medium (DMEM) supplemented with 10% fetal bovine serum was then added to keep hydrated. At cultivation day 2 and 5, cell-seeded scaffolds were removed and gently washed three times with PBS. The cells on the scaffolds were subsequently immobilized with 4% formaldehyde and osmium tetroxide at 4 °C for 15–30 min and washed gently with PBS for 3 times. The samples were then sequentially dehydrated with an ethanol series (50, 70, 90, 95, and 100%) 2 times each for 10 min. The samples were allowed to dry at critical point and coated with gold for SEM analysis of cell morphology and adhesion.

#### In vitro cytotoxicity assay

The 100 mg of square scaffold sheets (14 sheets, 7 mm × 7 mm × 1 mm/sheet) were sterilized and immersed in 20 mL medium at 4 °C for 48 h. Rabbit ASCs (5 × 10^4^ cells/mL, 100 μL) were seeded into 96-well plates and cultured in a 37 °C incubator for 48 h, and then the medium was refreshed. MTT (1 mg/mL, 100 μL, Sigma-Aldrich) solution was added to each well at day 1, 3, and 5 and incubated at 37 °C for 4 h. After removal of supernatants, 150 μL of dimethyl sulfoxide (DMSO, Sigma-Aldrich) was added to dissolve the blue formazan crystal. The absorbance was measured at 490 nm on an ELISA reader (Bio-Tek Instruments, Inc., Highland Park, VT, USA).

#### In vitro osteogenic differentiation

*In vitro* osteogenic effect of PLLA/nHA/CM-AL scaffolds was analyzed by alkaline phosphatase (ALP) activity assay and alizarin red staining. Rabbit ASCs (5 × 10^4^ cells/mL, 120 μL/well) were seeded into a 12-well plate containing extracted liquors from scaffolds supplied with or without osteogenic medium (OGM, DMEM, 10% FBS, 100 nmol/L dexamethasone, 0.05 mmol/L ascorbic acid, and 10 mmol/L β-glycerophosphate). ALP activity was measured on days 7 and 14 as described previously^31^. For alizarin red staining, the medium was replaced every 3 days. Cells were obtained on days 7 and 14 and fixed in 4% formaldehyde for 15 min. After washing twice with PBS, the samples were treated with alizarin red (0.5 mL, 40 mmol/L, pH = 4.1) for 25 min. The images were then taken under an inverted optical microscope (Nikon, Tokyo, Japan). The percentages of positive cells were calculated from at least 3 randomly selected fields.

### *In vivo* bone regeneration

This study was approved by the Ethical Committee of The Third Xiangya Hospital, Central South University (Changsha, China). All animal experiments were performed in accordance with the protocols approved by the Animal Care and Use Committee of The Third XiangyaHospital of Central South University. Rabbit radius defect model was established as described previously^52^. Briefly, New Zealand rabbits (weighing 2.6–3.4 kg), obtained from the experimental animal center of the Third Xiangya Hospital of Central South University (Changsha, China), were anesthetized with 3% pentobarbital. An incision was made to expose radius, and a 1.5 cm segmental bone defect was created on the bilateral radius of 24 rabbits. The defects created were filled in 9 rabbits with PLLA/nHA (n = 9, group III, 0%CM-ALs group) and PLLA/nHA/CM-AL (10%) (n = 9, group IV, 10%CM-ALs group) scaffolds immersed in dexamethasone and gentamicin mixture, respectively. The created defects in the other 6 rabbits were treated with either autologous orthotopic transplantation (n = 3, group II, autograft group) or without any treatment as blank control group (n = 3, group I). All animals were injected intraperitoneally with gentamicin (2 mL) for 3 days to prevent infection. X-Ray analysis was performed to evaluate bone formation at 2, 4, and 8 weeks after surgery. Results were quantified by ImageJ software. Three animals were sacrificed at each time points (2, 4, and 8 weeks) by air embolism for histopathological analysis. Left forelimbs were harvested and paraformaldehyde-fixed and paraffin-embedded specimens from bone defects were dissected for hematoxylin eosin (HE) and masson trichrome staining. Quantitative analysis of the sections was performed by imageJ with 6 sections from each sample.

### Statistical analysis

The data were expressed as mean ± standard deviation (SD). The experiments were performed in triplicates. Statistical comparisons were made by Student’s t test or one-way analysis of variance (ANOVA) in appropriate. P values of less than 0.05 were considered as statistically significant.
